# Supplementary material for: Trusting the experts: The domain-specificity of prestige-biased social learning
Source: PLoS One. 2021 Aug 11;16(8):e0255346. doi: 10.1371/journal.pone.0255346 (PMC8357104; doi:10.1371/journal.pone.0255346)

Supplementary Material for:

Trusting the experts: the domain-specificity of prestige-biased social learning

CONTENTS

Screenshots from experiment	pp1 – 6
Model specifications	pp6 – 7
Plots from exploratory analyses	pp. 7 – 8

Example screenshots for each stage of the experiment, and each Condition.

Please note, Round 1 is identical for each Condition.

Example of a question for the “Language” topic in **Round 1**. Players have two possible answers to choose from, or they can select “Ask Someone Else.” The number ‘8’ here is the countdown timer, telling players they have 8 seconds left to answer. Each player gets 15 seconds to decide on their first response.

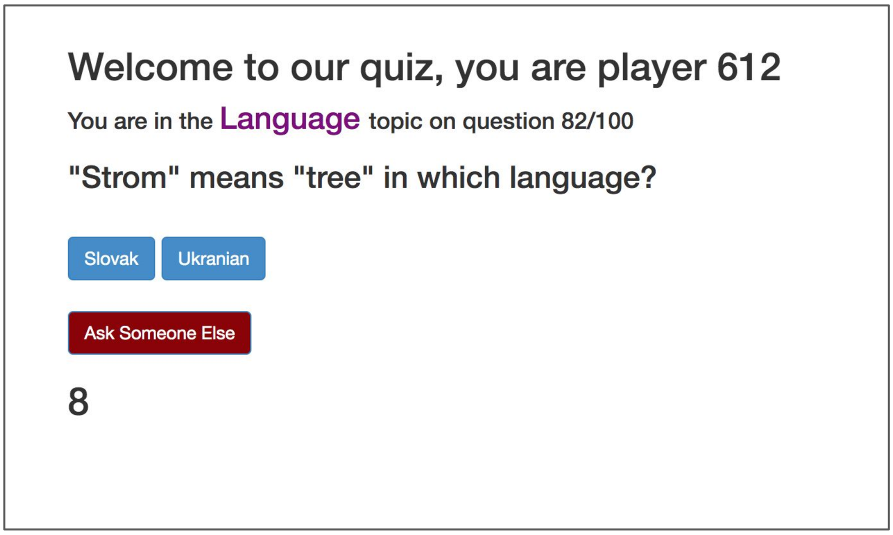

Example of **Round 1**, after choosing “Ask Someone Else.” On this question, 3 out of the other 9 participants answered for themselves. Those three player’s scores are displayed to each participant who chose to “ask someone else”, as below:

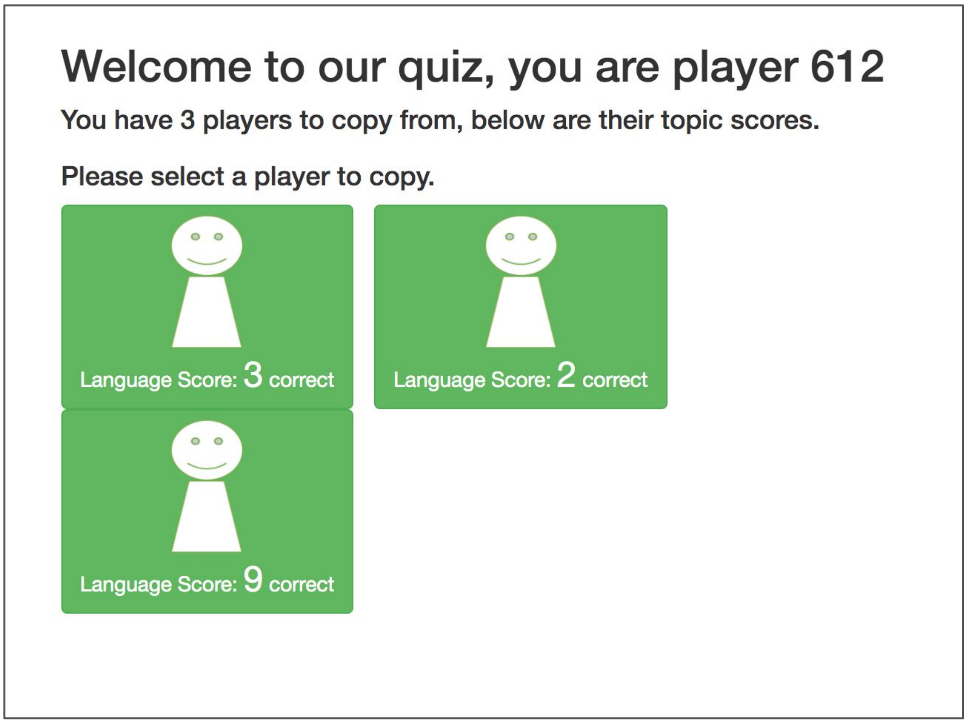

Example of for **Round 2, Condition A** after selecting “Ask Someone Else”

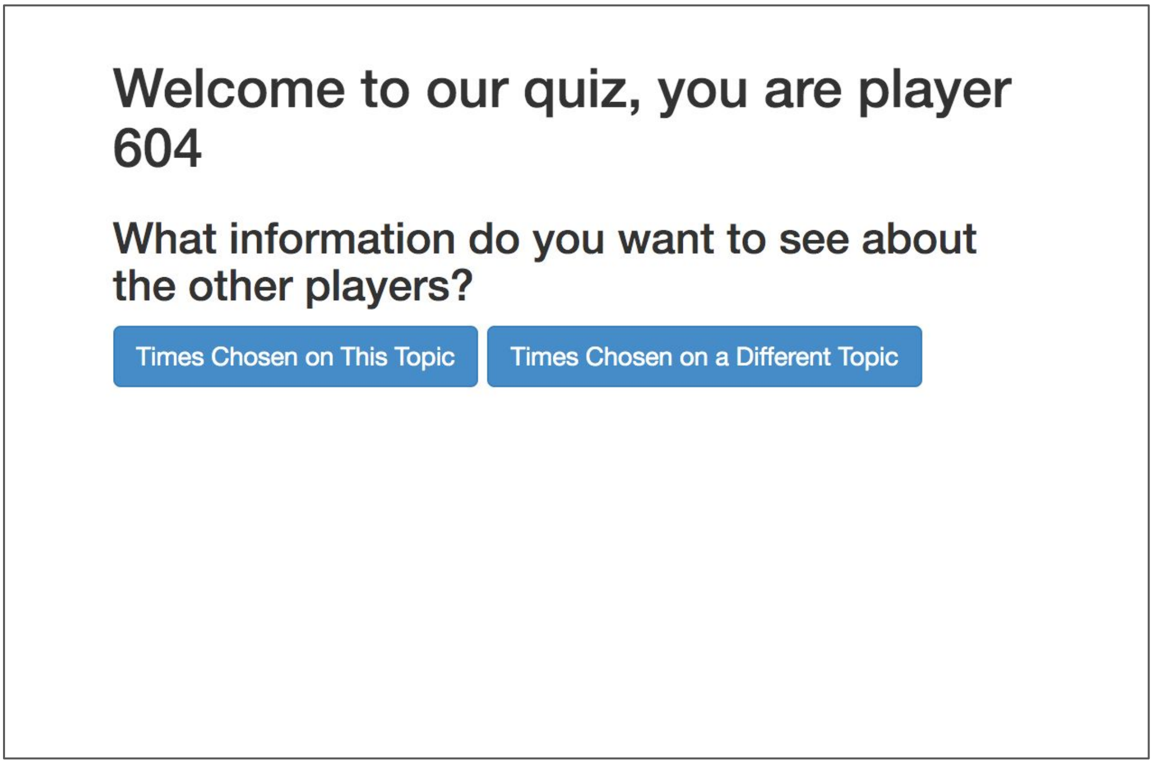

Example of **Round 2, Condition A** after choosing “**Times Chosen on a Different Topic”**

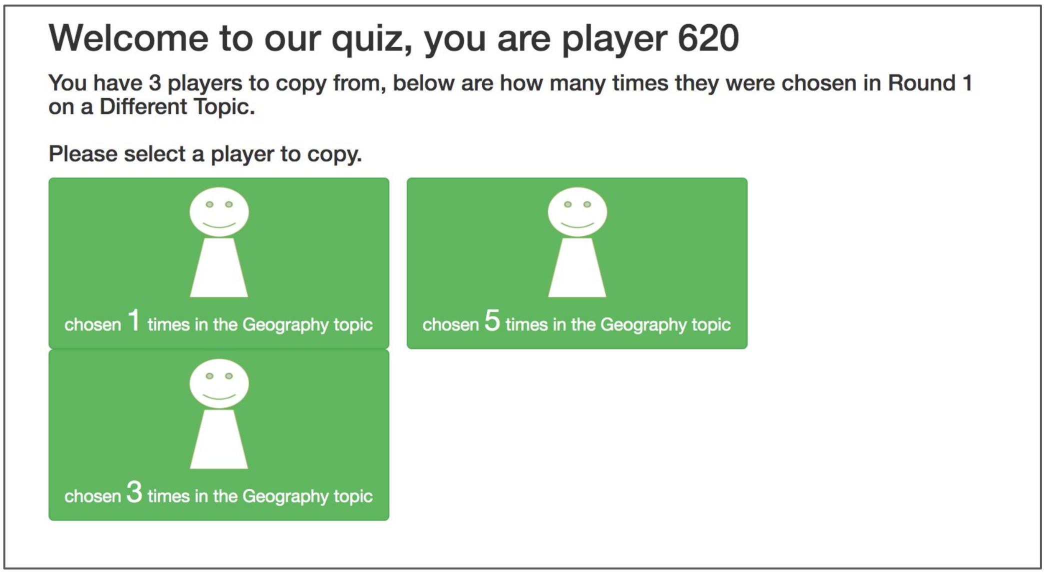

Example of for **Round 2, Condition B** after selecting “Ask Someone Else”

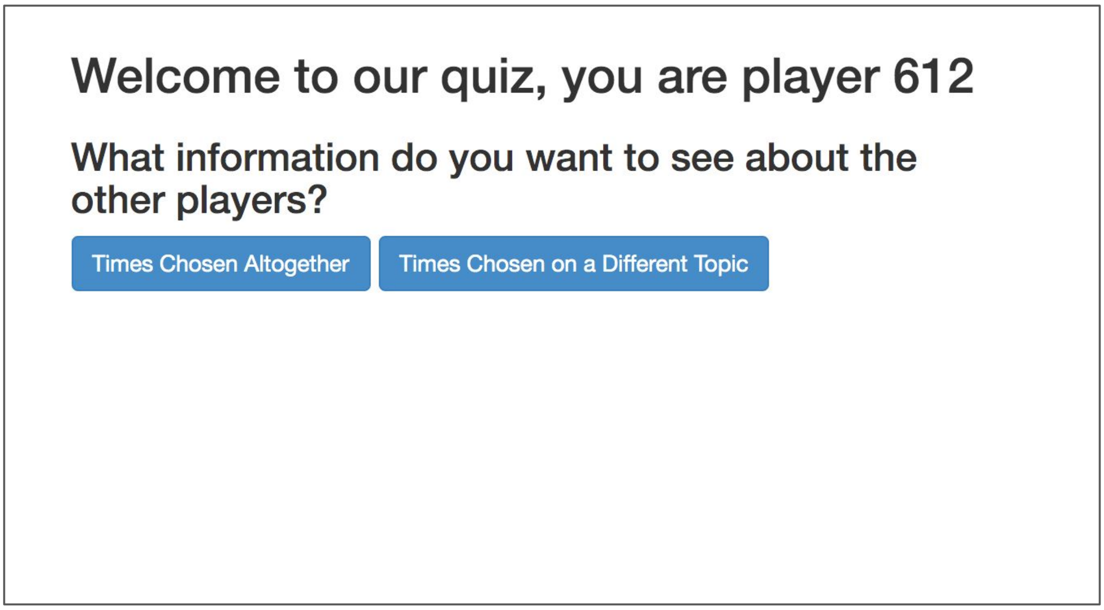

Example of **Round 2, Condition B** after choosing “T**imes Chosen Altogether”**

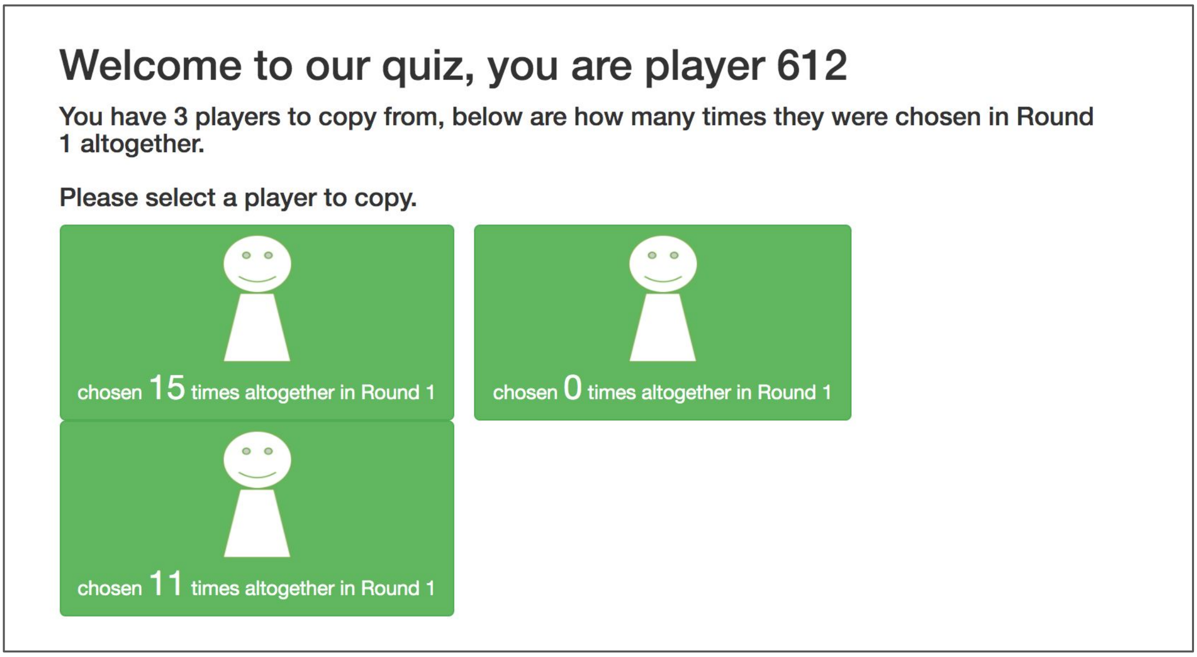

Example of for Round 2, Condition C after selecting “Ask Someone Else”

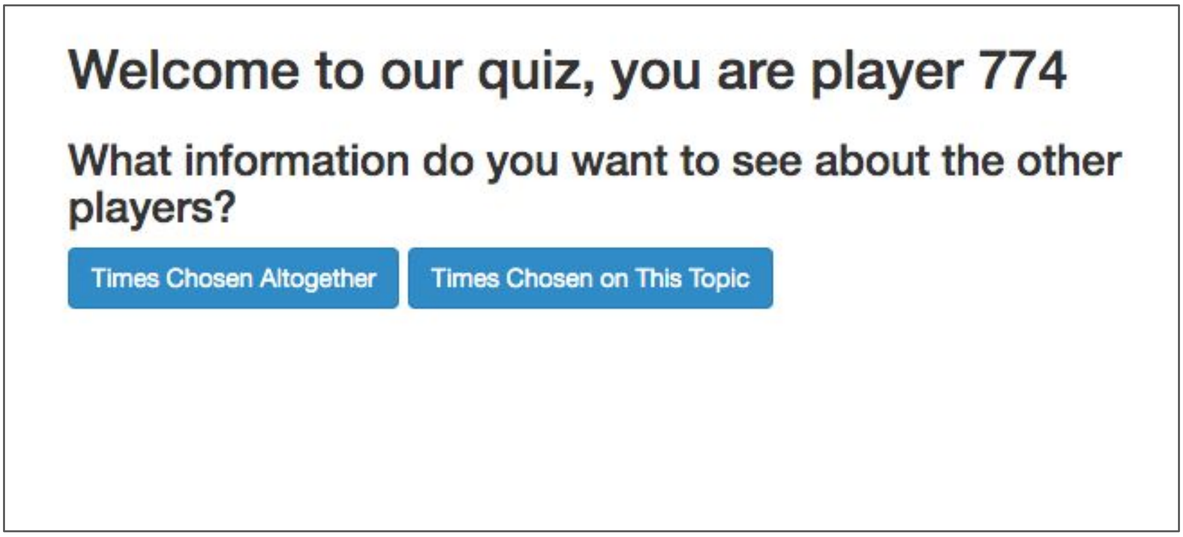

Example of Round 2, Condition C after choosing “**Times Chosen on This Topic**”

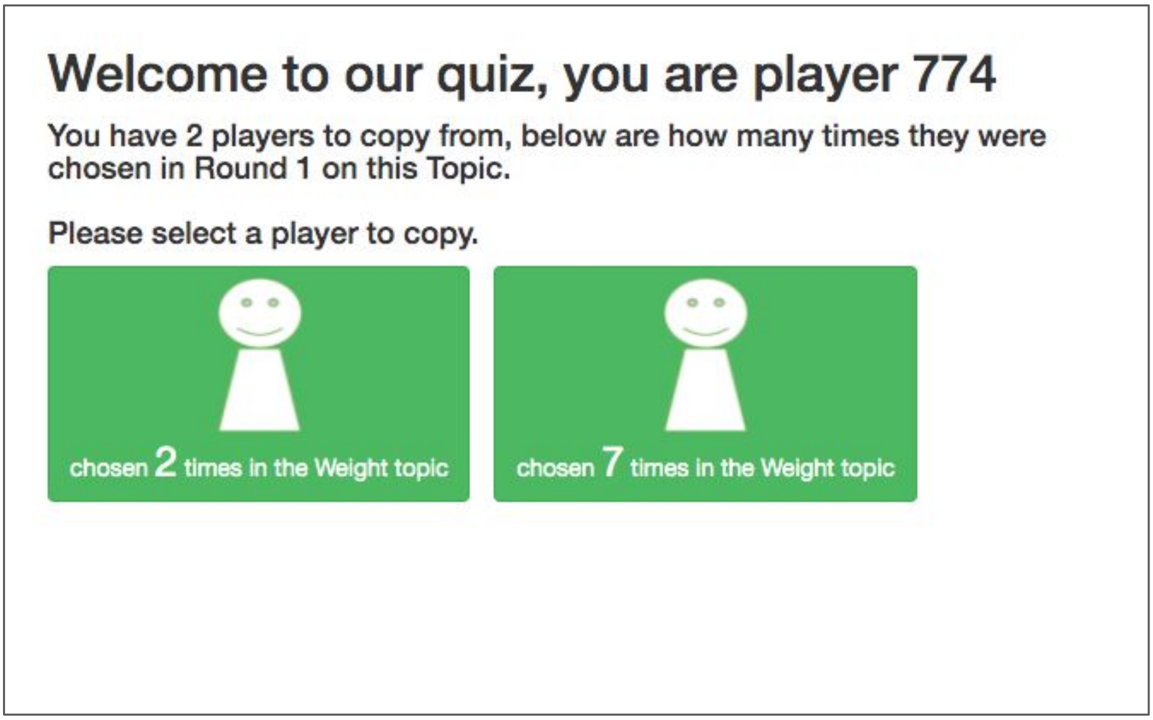

Example of for **Round 2, Condition D** after selecting “Ask Someone Else”

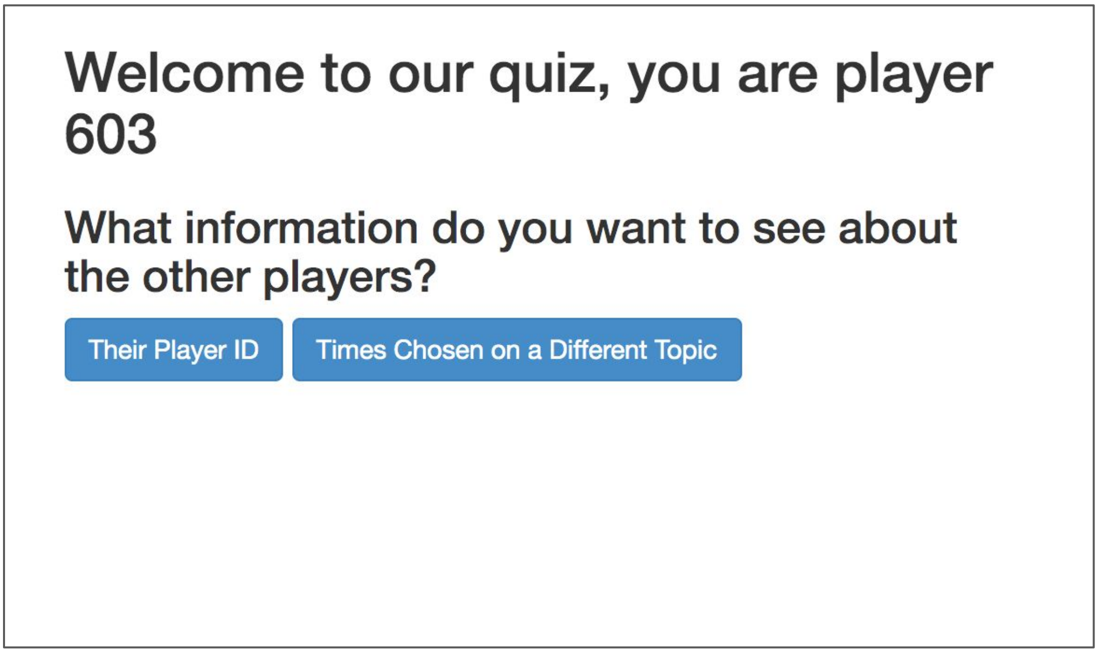

Example of **Round 2, Condition D** after choosing “Their Player ID”

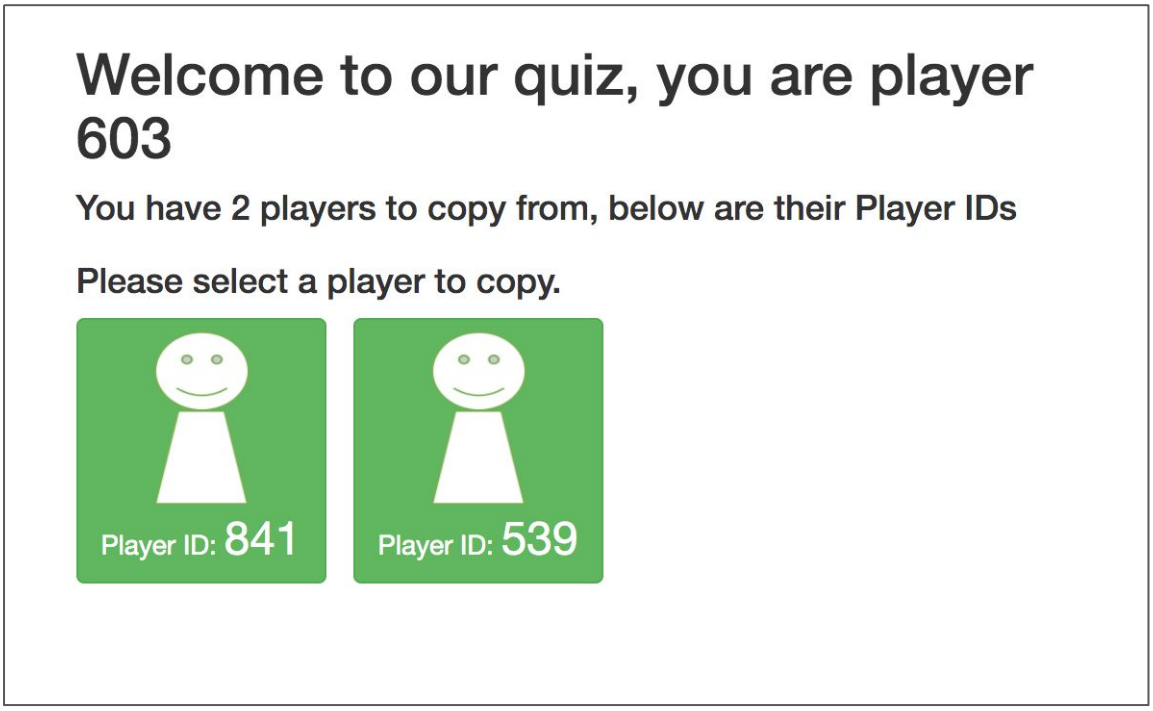

Model specifications

Full code and analysis scripts are available at https://github.com/lottybrand/Prestige_2_Analysis

All models were written according to the Statistical Rethinking course (McElreath 2016) using the *Rethinking()* package in R.

To test predictions 2 and 3 (whether participants chose to copy the highest scoring demonstrator available to them, and whether participants chose to copy the most copied demonstrator available to them), models 1 and 2 were used:

Model 1:

```
model1 <- map2stan(
  alist(
    copied_successful ~ dbinom(1, p),
    logit(p) <- a + a_p[pptIndex]*sigma_p + a_g[groupIndex]*sigma_g,
    a ~ dnorm(0,4),
    a_p[pptIndex] ~ dnorm(0,1),
    a_g[groupIndex] ~ dnorm(0,1),
    sigma_p ~ dcauchy(0,1),
    sigma_g ~ dcauchy(0,1)
  ),
  data=scoreChoice, constraints=list(sigma_p="lower=0", sigma_g="lower=0"),
  warmup=1000, iter=4000, chains=3, cores=3)
```

Model 2:

```
model2 <- map2stan(
  alist(
    copied_prestigious ~ dbinom(1, p),
    logit(p) <- a + a_p[pptIndex]*sigma_p + a_g[groupIndex]*sigma_g,
    a ~ dnorm(0,4),
    a_p[pptIndex] ~ dnorm(0,1),
    a_g[groupIndex] ~ dnorm(0,1),
    sigma_p ~ dcauchy(0,1),
    sigma_g ~ dcauchy(0,1)
  ),
  data=prestigeChoice, constraints=list(sigma_p="lower=0", sigma_g="lower=0"),
  warmup=1000, iter=4000, chains=3, cores=3 )
```

To test our main hypothesis (predictions 4, 5 and 6), that participants chose the information we prediction in each condition, model 3 was used:

Model 3:

```
model3.2 <- ulam(
  alist(
    chosePredicted ~ dbinom( 1 , p ) ,
    logit(p) <- a[pptIndex] + g[groupIndex] + b[condsIndex] ,
    b[condsIndex] ~ dnorm( 0 , sigma_b ),
    a[pptIndex] ~ dnorm( 0 , sigma_a ),
```

```
    g[groupIndex] ~ dnorm( 0 , sigma_g ),
    sigma_a ~ dexp(1),
    sigma_g ~ dexp(1),
    sigma_b ~ dexp(1)
  ) , data=infoChosen_list, constraints=list(sigma_a="lower=0", sigma_g="lower=0", sigma_b="lower=0"),
control=list( adapt_delta=0.99, max_treedepth=13),
  warmup=1000, iter=9000, chains=3 , cores=3 , log_lik=TRUE )
```

To test predictions 7 and 8 (whether participants copied more in conditions where domain-specific information was available, and whether participants scored higher in conditions where domain-specific information was available) we ran Models 4 and 5.

Model 4:

```
model4.2 <- ulam(
  alist(
    copied ~ dbinom( 1 , p ) ,
    logit(p) <- a_bar + a[pptIndex]*sigma_a + g[groupIndex]*sigma_g + b[condsIndex] ,
    b[condsIndex] ~ dnorm( 0 , 0.5 ),
    a[pptIndex] ~ dnorm( 0 , 1 ),
    g[groupIndex] ~ dnorm( 0 , 1 ),
    a_bar ~ dnorm( 0 , 1.5 ),
    sigma_a ~ dexp(1),
    sigma_g ~ dexp(1)
  ) , data=asocialOnly_list_2 , constraints=list(sigma_a="lower=0", sigma_g="lower=0"), control=list(
adapt_delta=0.99, max_treedepth=13),
  warmup=1000, iter=5000, chains=3 , cores=3 , log_lik=TRUE )
```

Model 5:

```
model5 <- map2stan(
  alist(
    t_score ~ dnorm(mu, sigma),
    mu <- a + b[condsIndex] + g[groupIndex],
    a ~ dnorm(50,10),
    b[condsIndex] ~ dnorm(0,0.5),
    g[groupIndex] ~ dnorm(0,0.5),
    sigma ~ dexp(1)
  ), data = finalScore_list, chains=3)
```

Exploratory analyses:

All code and data for exploratory analyses are available at:
https://github.com/lottybrand/Prestige_2_Analysis

In exploratory analyses we found that **more copying throughout the quiz does lead to a higher score on the quiz overall** (mean estimate: 0.24, 89%CI: [0.20, 0.28])
and that **higher asocial quiz score on the quiz overall does lead to a higher prestige score overall** (mean estimate: 0.22, 89%CI: [0.14, 0.30]).

The below plot shows the relationship between asocial quiz score and prestige score, comparable to previous results (Brand et al. 2020).

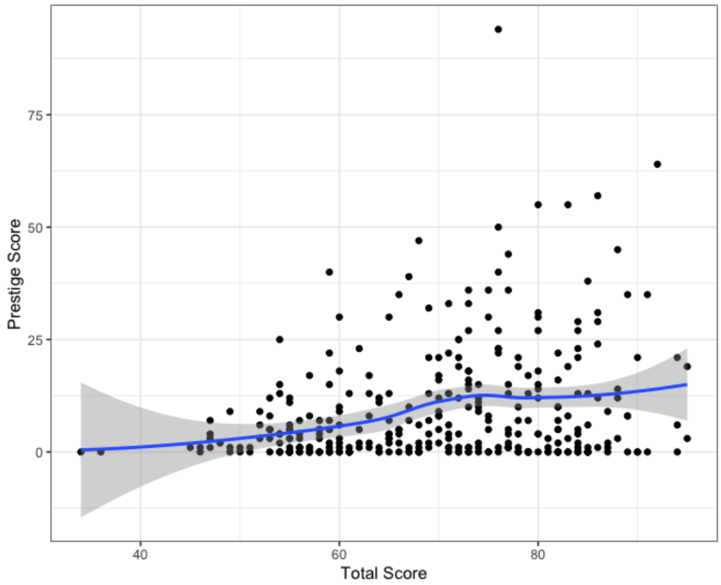

The below plot shows the individual differences in prestige score, comparable to previous results (Brand et al. 2020). This shows that, the vast majority of participants were never copied, or copied under 20 times, but that a handful of participants became extremely prestigious in their group, being copied over 40 times, in each condition except Condition D.

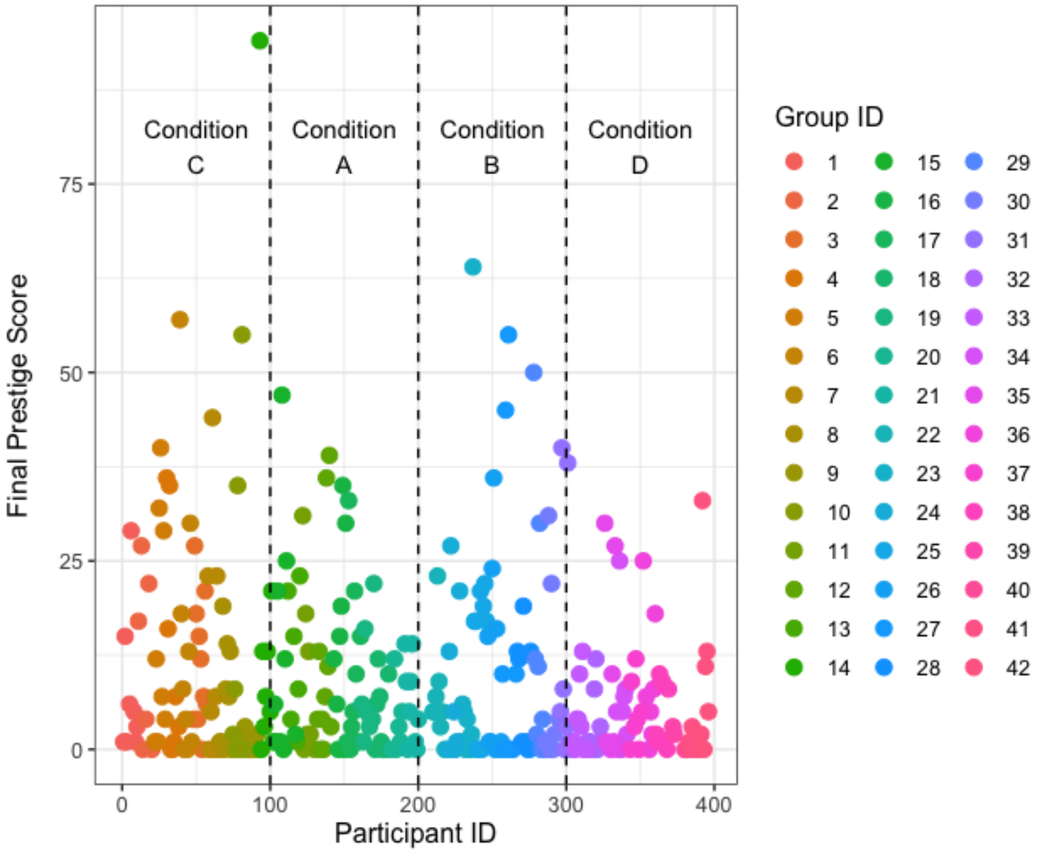

Supplement: S1 File — (PDF) [file pone.0255346.s001.pdf]
