# Supplementary material for: Trusting the experts: The domain-specificity of prestige-biased social learning
Source: PLoS One. 2021 Aug 11;16(8):e0255346. doi: 10.1371/journal.pone.0255346 (PMC8357104; doi:10.1371/journal.pone.0255346)
Supplement: Attachment — Submitted filename: response_to_reviewers_rev3.pdf [file pone.0255346.s003.pdf]

PONE-D-20-39514R1
Trusting the experts: the domain-specificity of prestige-biased social learning
PLOS ONE

Dear Dr. Brand,

Thank you for submitting your manuscript to PLOS ONE. After careful consideration, we feel that it has merit but does not fully meet PLOS ONE's publication criteria as it currently stands. Therefore, we invite you to submit a revised version of the manuscript that addresses the points raised during the review process.

Thank you for seeking further reviews on our manuscript and inviting us to submit a revised version. We hope the below responses and additional changes to our manuscript address all concerns. Please find our response to comments in bold below.

==============================

Below you will find the comments provided by Reviewer 2 and a new reviewer (4). Reviewer 1 declined the invitation to read your manuscript again. As you can see, Reviewers 2 and 4 make radically different recommendations: while Reviewer 2 now recommends acceptance, Reviewer 4 recommends outright rejection. My own impression is that the concerns raised by Reviewer 4 (partially overlapping with those of Reviewer 1) do obscure the interpretation of the results and merit, at least, a detailed discussion in the main text. In the same vein, I think that some of the problems previously detected by Reviewer 1 should lead to further changes in the text. Both Reviewers 1 and 4 note that the fact that you didn't manipulate prestige cues experimentally undermines your interpretation of the results. This is perhaps clearest in the new review provided by Reviewer 4. The main concern is that because during Round 1 participants only had information about the domain-specific score of the other participants, then all measures of prestige in Round 2 can be seen as proxies for the domain-specific score of the other participants. That is, the information that participants see in Round 2 about who was imitated most often in Round 1 must be determined by the domain-specific scores in Round 1. This obscures whether the results seen in Round 2 are actually driven by prestige cues or rather by inferred domain-specific accuracy. In your response to Reviewer 1 you argue that your procedure provides a more natural test of how prestige dynamics arise in the real world. This is a fair point, but I think it deserves an explicit discussion in the main text. It is absolutely fine to emphasize the advantages of using a naturalistic procedure, but the reader should also be alerted about the potential problems of this strategy.

Both reviewers 1 and 4 are correct that the prestige cues in our experiment are proxies for the domain-specific score of individuals. However, this is by design, as this is precisely how prestige is defined in the cultural evolutionary theory of prestige *(Henrich, J., & Gil-White, F. J. (2001) The evolution of prestige: Freely conferred deference as a mechanism for enhancing the benefits of cultural transmission. Evolution and human behavior, 22(3), 165-196)*. Because this is the theory that our work aims to test, we adopt its definitions, as we did in previous related work (*Brand, C. O., Heap, S., Morgan, T. J. H., &*

***Mesoudi, A. (2020) The emergence and adaptive use of prestige in an online social learning task. Scientific reports, 10(1), 1-11).* Nonetheless, we recognise that definitions of prestige vary across fields and so we have edited the manuscript to make the exact definition we are working with more explicit, both in the introduction (lines 60-83) and the discussion (lines 506-509).**

It is true that our experiment is both naturalistic (in that we are not providing artificial or manipulated prestige cues to the participants but letting them emerge from their behaviour) and "unrealistic" (in that participants have direct access to participant success in the first round). However, all experiments contain both naturalistic and unrealistic elements; an experiment that contained no connection to the real world would tell us little, but equally, the whole point of experiments is to unrealistically manipulate variables and conditions. We think that our combination of endogenous prestige cues plus manipulated access to success provides a powerful way to test the aforementioned prestige theory from the cultural evolution literature. This is now explicitly highlighted in the manuscript (lines 143-148). Note also that this paradigm has been previously used and is discussed in more detail in those publications *(Brand et al. 2020)*.

We have also responded to these points more thoroughly in response to Reviewer 4 below.

If I understand the design properly, condition D was not part of the original preregistered protocol and was added after data were already collected for conditions A-C. An unfortunate consequence of this is that all the analyses that include condition D fall outside the scope of the pre-registered protocol. Please note this in the main text (i.e., that all models involving condition D depart from the protocol). Also, condition D is introduced in Table 1, but the fact that this condition was not part of the preregistered protocol is not explained until line 450. Please, explain as early as possible in the ms that condition D was not part of the preregistration.

This is a misunderstanding, caused by lack of clarity in the manuscript which we have now addressed. Condition D *was* part of the pre-registration: we were always going to run a fourth condition based on the outcome of our first three conditions. As such, the details of Condition D are included in the pre-registration (page 7 under "Follow-Up Analysis"), with the specification that we would run it based on whichever information was *least preferred* out of Conditions A-C. This is already detailed in the manuscript methods section headed "Unregistered predictions" (lines 378-394), which preceded the analysis section in the previous version of the manuscript. Due to the reordering of the analysis section it may have been less clear, so we have now made this more explicit whenever mentioning Condition D throughout the methods, including Table 1 (lines 260, 271- and 379-394). It is worth noting that we also highlight here that our assumption check wording differs from the preregistration wording for prediction 1, as we realised during analysis that we wanted this analysis and assumption check to be consistent with our previous study (lines 298-301).

I understand that conditions A-D were manipulated between participants, but I don't think this is clearly stated anywhere in the manuscript. Please, say so explicitly and consider referring to Groups A-D instead of conditions A-D. Note that this is also relevant to

understand Reviewer 4's concerns about your mixed models (i.e., whether participants are nested within groups or crossed).

There were ten groups of ten participants *within* each Condition, so the four Conditions (A, B, C, D) each had ten groups of interacting participants within them. This is reflected in the structure of our statistical model (which we have clarified in response to Reviewer 4 below), and we have clarified the general experimental design in the methods section (lines 194-196.)

Reviewer 1 complained that the general procedure was difficult to follow and suggested adding a figure. I think that the new figure 1 does help the reader understand the procedure, but it would be even better to present not only a trial from one particular condition in Round 2, but examples also from Round 1 and the remaining conditions in Round 2.

We understand this concern, and have added further figures in the supplementary material. Including each decision for each condition in both rounds would result in 16 figures altogether, which would make the main manuscript quite busy. Rather than duplicate Figure 1 three more times, we have provided an example of each Round 2 decision in the supplementary material (Round 1 decisions are the same for all Conditions).

Reviewer 1 also questioned the adequacy of Figure 3. I do share the feeling that the information conveyed by Figures 2 and 3 is somewhat redundant.

Figure 2 contains the raw data which displays how many copying instances occurred in each condition, as well as which information was chosen. This allows the reader to see that the amount of copying differed between conditions, as well as which information was preferred. Figure 3 displays the model predictions based on statistical modelling, confirming that participants did indeed show a very strong preference for the information type that we predicted in each condition, whilst controlling for variation within individuals, within groups, within question, and within conditions, which we cannot know from looking at the raw data only. Therefore we would not be comfortable removing either figure as both provide crucial and separate information for the reader to make appropriate inferences themselves.

Reviewer 1 asked for some justification of sample size. Although your response is compeling it is true that in the present version of the ms it is difficult to appreciate whether your sample is sufficiently sensitive given the effects you are studying. I don't think a power analysis would be appropriate here (because you are not using frequentist stats), but it would be nice to have some measure that allowed the reader to infer whether the number of observations is large enough to conclude with certainty that the effects you find are conclusively different from zero (or not). I think that Bayes factors against the null hypotheses would serve this purpose well.

We agree that it is important to make clear whether we can reach conclusions with certainty, however, the provided results already achieve this. Specifically, for parameter estimates we provide credible intervals which are derived from the posterior distributions

and identify which values we can and cannot rule out (this is in contrast to frequentist confidence intervals). As such, where these intervals exclude 0 this corresponds to being able to conclude with certainty that the effect is different from 0. Bayes factors also serve this purpose, but adopt a different approach (model comparison/hypothesis testing as opposed to parameter estimation). Thus, to include both would amount to duplication of the same analysis.

We are taking the approach recommended by McElreath's "Statistical Rethinking" course *(McElreath, R. (2018). Statistical rethinking: A Bayesian course with examples in R and Stan. Chapman and Hall/CRC)*, which emphasises estimation and communicating uncertainty rather than Null Hypothesis Significance Testing and arbitrary cut-offs; for this reason we are reluctant to use Bayes Factors to achieve a "yes/no" answer when we are already communicating results along with their uncertainty for the reader to interpret themselves. We note that Reviewers 2, 3 and 4 commended the transparency and strength of our statistical analyses and results.

It's also worth noting that our main analysis of interest was based on participant's copying choices. As participants could copy as many or as few times as they liked, we were unable to perform a power analysis in advance to account for how many or how few times participants would choose to copy in the experiment, as the decision to copy or not was down to each individual in each question. We therefore collected ten groups of ten for each condition to ensure that enough copying instances would occur, resulting in 1,947 copying instances (out of a possible 40,000 instances if all 400 participants copied on all 100 questions). Our model structure accounts for the fact that choices are clustered within questions, participants, groups and conditions, and so pseudoreplication is not a problem with this analysis, and our actual sample size is therefore 1,947 - notably an order of magnitude larger than the majority of psychological research on decision making, which often does not account for pseudoreplication in the analysis.

Reviewer 1 complained that the analytic strategy was also difficult to follow. I do think that this version is clearer but it would be even better to merge completely the paragraphs where you explain the predictions and the paragraphs where you explain how you will test them. In other words, explain on lines 380-385 that you will test prediction 1 using correlation coefficients. Merge lines 395-401 with Prediction 2 and in Prediction 3 simply explain that the analysis will be as in Prediction 3; and follow the same logis with the remaining predictions and analysis plans. Otherwise the reader is constantly forced to go back to each prediction when reading the proposed analyses.

Thank you for this suggestion, we have now reordered these sections as you suggest.

Minor comments:
line 60: "Here, 'bias' in meant..." -> "in" should be "is"

Thank you for spotting, this typo has now been corrected.

line 141: Double space after "four"

Thank you, we have now removed this.

lines 460-472: on a first read, it is unclear whether this refers to all the analyses or just to the one immediately above. Please clarify this here.

Apologies, but the number lines do not correspond to the manuscript version that was submitted (domain_specificity_prestige_bias_rev2.docx) nor the tracked-changes version, nor the final pdf. The other line numbers you mentioned in this section were off by a few, so I was able to track down the part you meant, but I cannot seem to track down which 12 lines this refers to unfortunately.

table 2: please report confidence intervals (or credibility intervals) for these correlation coefficients, so that the reader can appreciate to what extent the numerical differences between them are meaningful or not.

Thank you, we have now added these to Table 1.

Starting on line 568 you report for each the model intercepts and refer to them as "mean coefficient estimate". The reader is forced to go back to the model description to understand that this is an intercept. Please, refer to this as mean intercept estimate instead or alert the reader somehow what these numbers mean.

Thank you, we have now adjusted the wording as you suggest.

lines 695-697: The second part of the sentence (i.e., "... and emerge either through unconscious associative learning, conscious deliberation, or both") doesn't add information beyond what's already said in the first half. It simply says that the nature of this type of learning is unknown.

We have adjusted the wording of this sentence, but feel that the second half does add information. We are not trying to say that the type of this learning is unknown, but that this learning likely emerges through either unconscious associative learning, or conscious deliberation, but probably a combination of both.

==============================

Reviewer #2: The authors of the manuscript "Trusting the experts: the domain-specificity of prestige-biased social learning" have successfully answered the comments I raised in the previous version.

Thank you very much again for taking the time to critically read our manuscript.

I would still try to make clearer the relationship, if any, between prestige and expertise but this is only a suggestion that in no way modifies my position to accept the manuscript as it stands.

We hope we have clarified this point further now, given the requests of the other reviewers also.

Reviewer #4: In the experiment reported in this manuscript, participants were given the opportunity to choose to answer each one of 100 quiz questions (in two rounds) by themselves, or copying from another participant. In the different conditions of the experiment, the available sources to copy from in the second round of questions were manipulated (using different pairs of sources in each condition) in such a way that participants could choose between two sources with different types of prestige (within-field, between-fields, general, or a random cue). The authors conclude that any type of prestige cue drives decisions (is preferred) relative to no prestige, within-field relative to general and between-fields, and general to between-fields.

As a general comment, I commend the authors for the transparency in performing and reporting the study. Preregistration, and sharing data and code, as done here, is a warranty that the authors are not using analysis flexibility to surreptitiously manipulate results in their favor. Moreover, the theoretically relevant effects are probably strong enough to provide substantial evidence in favor of all the main hypotheses, regardless of the statistical approach used.

Thank you very much for acknowledging our transparency, the theoretical relevance of our effects, and the strength of our evidence.

Still, the preregistered plan describes the model to test main hypothesis as a Bayesian GLME with the following structure: Chose_predicted ~ intercept + 1|condition + 1|Participant + 1|group +1|topic

If this is the model that was finally run (I am sorry I am not familiar with the specific syntax of the package used for Bayesian analysis here), why is condition modelled as a random-effects factor (random intercept)? As far as I know, random-effects factors are those for which levels can be considered as randomly sampled from the set of possible ones, whereas here levels are actively manipulated. And also, this syntax suggests that group and participant factors were crossed, but in the design participants are actually nested in groups. Please clarify these issues, or correct me if I am misinterpreting something.

This is a common interpretation of syntax used to represent a frequentist glmm, stemming from the lmer() package. However it is not equivalent for representing our model, which uses the Rethinking() package. Our model does not differentiate between cross or nested factors in the same way. Moreover, the use of varying intercepts for treatment effects (i.e. conditions) is statistically appropriate, despite common concerns. See page 423, section 13.3.2 of Statistical Rethinking, McElreath 2020: “You might notice

that the treatment effects (i.e. Condition effect), look a lot like the a and g parameters (i.e. Participant and Group effects). Could we also use partial pooling on the treatment effects? Yes, we could. Some people will scream "No!" at this suggestion, because they have been taught that varying effects (i.e. "random effects") are only for variables that were not experimentally controlled. Since treatment was "fixed" by the experiment, the thinking goes, we should use un-pooled "fixed" effects. This is all wrong. The reason to use varying effects is because they provide better inferences. It doesn't matter how the clusters arise. If the individual units are exchangeable— the index values could be reassigned without changing the meaning of the model—then partial pooling could help." Here, our Condition effects are numbered 1 - 4; their index values are exchangeable, as it wouldn't matter if the Conditions were numbered differently, the interpretation of their effects would be the same, just as the numbering of participants, groups, or questions.

My most important concern, however, is not methodological but conceptual. The authors claim that prestige emerges from the task, but I am afraid the very concept of prestige is totally unnecessary to account for the results.

As detailed in the manuscript (and in the authors' response to a reviewer from the previous round), participants had access to the in-field accuracy of answers from the other participants in the first round. It is true that participants do not have access to information on whether peers also tended to choose the responder with the highest number of correct answers, but assuming that "most people will do as I do" seems rather straightforward to me. In other words, the task is not letting prestige "emerge", it is just providing an almost perfect proxy (the most frequently chosen responder) to in-field objective accuracy.

Consequently, if, as a responder, I have a perfect proxy to in-field accuracy I also have a less perfect proxy to general accuracy (as in-field accuracy mathematically contributes to general-accuracy), and an even less perfect proxy to between-fields accuracy. That is, the ordering of preferences in the second round does not require to assume the use of any prestige cue at all, but just plain reasoning based on estimated accuracy.

At the present moment, I see no necessity for prestige as an intermediate explanatory construct. As mentioned by one of the reviewers in the previous round, it would have been more convincing to actively manipulate prestige cues. Hence, unless I am wrong here (and I hope I am, given the carefulness with which this study has been carried out), my recommendation will be not accepting this manuscript for publication.

Your characterisation of our experiment is correct in that participants had access to accuracy in the first round, and hence they have a direct proxy to accuracy in the second round. However, this is intentional, as we are testing a direct prediction from the theory of how prestige evolved, put forward by Henrich & Gil-White in 2001 *(Henrich, J., & Gil-White, F. J. (2001) The evolution of prestige: Freely conferred deference as a mechanism for enhancing the benefits of cultural transmission. Evolution and human behavior, 22(3), 165-196)*. This work assumes that prestige arose as a result of using proxies of success as a shortcut to having to evaluate actual success. The most obvious and direct proxy of success initially would be whether other members of the group are copying this person.

Thus "more attention" or "admiration" are what we call "prestige cues." Thus "number of times copied" in our experiment is the most basic and immediate prestige cue that we might expect in generating prestige hierarchies from which prestige-biased social learning emerges. This is precisely how prestige is defined in the cultural evolutionary theory of prestige (Henrich & Gil-White 2001). Because this is the theory that our work aims to test, we adopt its definitions, as we did in previous related work *(Brand, C. O., Heap, S., Morgan, T. J. H., & Mesoudi, A. (2020) The emergence and adaptive use of prestige in an online social learning task. Scientific reports, 10(1), 1-11)*. Nonetheless, we recognise that definitions of prestige vary across fields and so we have edited the manuscript to make the exact definition we are working with more explicit, both in the introduction (lines 60-83) and the discussion (lines 506-509).

Whilst other work on prestige-biased social learning has manipulated or created artificial prestige cues in their design, we intentionally did not do this, as it starts from the assumption that those cues (e.g. attention, admiration, respect) arose based on success in the first place. The literature was lacking any formal test of this basic assumption, and so our initial design was to precisely test whether 'prestige cues' such as 'attention' can arise as a consequence of wanting to copy the most successful, but not having access to this information directly. Therefore the "number of times copied" can be interpreted as "attention by other group members." This was the aim of our previous experiment that tested this experimental paradigm, and there is much more discussion of this paradigm in the previous paper (Brand et al. 2020). This previous paper also addresses your concern that our experiment relies on the assumption that "most people will do as I do" and "just plain reasoning based on estimated accuracy," as in fact this is not always the case, and is an empirical question that needs testing rather than can be assumed of all human behaviour. Our previous experiment was based on a pilot experiment in which the "random cue" in the control condition was participants' hobbies, rather than their randomly generated ID number. Their hobbies obviously had no relation to their success on the quiz, nevertheless many participants chose to view this information *instead of direct score on the quiz*, which we would not expect based on "plain reasoning based on estimated accuracy" and is certainly not most people "doing as I do" when trying to achieve the highest possible quiz score. Again, more discussion of these points are included in our previous paper that this work builds upon (Brand et al. 2020).

It is true that our experiment is both naturalistic (in that we are not providing artificial or manipulated prestige cues to the participants but letting them emerge from their behaviour) and "unrealistic" (in that participants have direct, free access to participant success in the first round). However, all experiments contain both naturalistic and unrealistic elements; an experiment that contained no connection to the real world would tell us little, but equally, the whole point of experiments is to unrealistically manipulate variables and conditions. We think that our combination of endogenous prestige cues plus manipulated access to success provides a powerful way to test the aforementioned prestige theory from the cultural evolution literature. This is now explicitly highlighted in the manuscript (lines 143-148). Note also that this exact paradigm has been previously used and is discussed in more detail in that publication (Brand et al. 2020).
