# Supplementary material for: Trusting the experts: The domain-specificity of prestige-biased social learning
Source: PLoS One. 2021 Aug 11;16(8):e0255346. doi: 10.1371/journal.pone.0255346 (PMC8357104; doi:10.1371/journal.pone.0255346)
Supplement: Attachment — Submitted filename: response_to_reviewers_.docx [file pone.0255346.s002.docx]

Dear Dr. Brand,

Thank you for submitting your manuscript to PLOS ONE. After careful consideration, we feel that it has merit but does not fully meet PLOS ONE’s publication criteria as it currently stands. Therefore, we invite you to submit a revised version of the manuscript that addresses the points raised during the review process.

As you will see, all the reviewers see merit in your study and provide overly positive reviews. Reviewer 3 recommends accepting the present version as is. Reviewers 1 and 2 in contrast suggest a number of revisions. In particular, both reviewers think that the the readability of the paper can be improved, particularly in the Methods and Results sections. I share their concerns (it was also difficult for me to understand those sections of the paper on a first read) and encourage you to take their suggestions to the heart. In addition to the detailed analytical recommendations made by Reviewer 1, I'd suggest reporting raw correlations (instead of R2) as a measure of association between variables, because they are substantially easier to interpret for most readers (R2 are absolutely fine as a measure of model fit, but I think they can be misleading in this context).

Thank you for taking the time to carefully read our manuscript. We hope our revisions have now made the methods and results section clearer. We’ve also altered the reporting of the correlation coefficients, as well as other substantial clarifications required by the reviewers below.

Reviewers' comments:

**Reviewer #1:** This manuscript reports one experiment in which the preferences for different prestige cues are compared, in the context of a quiz game. The authors conclude that prestige drives the tendency to choose who must be copied during the game. Additionally, domain-specific prestige is preferred to cross-domain and domain-general prestige, whereas domain-general prestige is preferred to cross-domain prestige, thus revealing a hierarchy of prestige sources.

In general, I think that the manuscript has merit. The research question is sound, and the methods seem adequate to address the question. The writing is overall clear, although I had some difficulties in understanding the analyses.

Thank you for taking the time to thoroughly read and engage with our work. We really appreciate your comments and recommendations.

What follows is a list of more specific comments on the article.

-First of all, I do not understand well why the phenomenon studied here (relying on prestige cues to choose who to learn from) is presented as a “bias”. It could certainly be understood as a simple rule, or heuristic. However, as far as I know, the term bias conveys (perhaps implicitly) the idea of systematic error or departure from a normative standard. But, how can we determine the standard (unbiased) behavior in a task like this? Is trusting individuals according to their prestige more biased than using their performance as a cue? In fact, it seems like a reasonable rule that should lead to good decisions most of the time (the Introduction describes the “bias” as “adaptative”). I think this needs further comment and clarification, perhaps in the Introduction.

Thank you for raising this important point that has been raised by others outside of the field of cultural evolution. We use the term ‘bias’ in the social learning literature as a shorthand for systematically departing from random sampling or random copying (‘unbiased copying’). This is different to how the term is often used in psychology as departing from a norm or containing errors. We have now added a section in the introduction to make this clearer (lines 58 – 65)

-I appreciate the transparency in the reporting of data and results (sharing data and code, pre-registering hypothesis).

Thank you.

-Although the article is generally clear, I found it difficult to follow in the Analyses/Results section. I am not sure that the way the manuscript is organized actually helps. On the one hand, presenting the predictions and the analyses schematically, before the results, seemed useful the first time I read it. However, in this study, we have different analyses with different dependent variables (if I understood well: probability of choosing the predicted option, copying rate, and quiz score). When we read the Results section, it is easy to forget which variable we are talking about, or what “prediction 3” (explained before) means. I needed to go back and forth several times to understand what the analyses were actually doing. I suggest to improve this by either organizing the text differently, or by adding explanations so that we do not need to go back and read the predictions/analysis plan. Perhaps organizing this section by dependent variables could help (i.e., first we analyze one variable, and when we finish we start with the next one, and we always include comments to remind the purpose and interpretation of the analysis), but I’m not sure.

Thank you for raising this, we did test a lot of predictions with this experiment and so we can see how it is difficult to follow the analysis/results section, we have reorganised and re-labelled the analysis and results section to hopefully make this easier to follow, by merging the prediction and analysis sections, and labelling the results to more easily link back to each type of prediction.

-When interpreting Figure 3, I wonder about the usefulness of this analysis, which in addition is quite complex to understand. The dependent variable is the probability of choosing the predicted option. Thus, the higher the model estimates, the better the researchers’ predictions were. This is a transformed variable that is not easy to interpret (note that in one condition, choosing “Domain-general” is coded as 1, but in other conditions the same answer is coded as 0). For example, how should we interpret the lack of differences between conditions in this variable? If you had found a difference in one condition against the others, how would you interpret it? I am not sure that the statistical model that is used here is actually useful. At least, I would need the authors to interpret the results when they are presented so that I can fully understand what the analysis reveals. Otherwise I had the impression that the comparison between conditions is meaningless, but I could be wrong.

You are correct that the comparison between conditions is meaningless, however, our results do not rely on such comparisons. Rather, our pre-registered predictions concern behaviour *within* (as opposed to *between*) conditions (except for predictions 7 and 8, see below). As such, Figure 3 demonstrates that the data supports our predictions for each condition, but we do not attempt any comparisons between the conditions as these are not relevant to our predictions. We now make this clear in the manuscript as we understand how it might seem misleading to present the model outputs for each condition in the same figure, whilst not interpreting differences between conditions. For predictions 7 and 8, where comparisons between conditions are relevant, we make this clear. We hope that renaming and relabelling these sections makes it easier to follow.

-I also found the design a little bit overcomplicated, and this may be due to the procedure used. First, experiments need manipulation and control of variables. Here, the only manipulated variables are the two options that are presented to each participant. The rest of parameters (the participant’s score, the participants’ prestige...) are uncontrolled, as they are generated spontaneously by participants (do I understand well the procedure?). If this is an experiment and we have clear predictions as to which cues should play a role, why not manipulating them directly? This could be achieved easily. For example, imagine that participants are presented a list of potential players to copy. But instead of real players (i.e., other participants) whose scores and parameters are beyond the control of the experimenter, you show them a list of fictitious players whose parameters are fixed by the experimenter (e.g., Player 111 has been copied 3 times in this domain, 5 times in another domain, etc.). Thus, you could manipulate the variables while controlling for other factors. The consequence of surrendering this degree of control is that the data we obtain are weaker, as we do not know whether the performance of the participants is actually affecting their individual decisions to copy/not copy, etc. What happens if, by chance, your 10 participants are very good at Geography and nobody asks for help? This affects the prestige cues that are crucial to your conclusions. At least a clarification about why the current procedure was preferred and what the benefits are is due.

We understand the desire to manipulate prestige cues directly, and previous experiments have attempted this. However we feel there is merit to letting prestige arise as a product of participant’s natural learning behaviour, as a more powerful test of how prestige dynamics may arise and vary naturally, rather than artificially constraining these parameters. In this regard we feel the data is a stronger test of predictions, not weaker. Moreover, avoiding manipulating stimuli like prestige cues means we do not need to deceive participants at any point (or inform them that cues have been manipulated).

To answer your specific question ‘what if, by chance, your 10 participants are very good at Geography and nobody asks for help’? We hoped to account for this chance happening by having 100 participants take part in each condition. Prior experiments involving similar designs find a moderate rate of copying and this informed out expectation. As it happened, this was borne out (we had between 200-700 copying instances in each condition, out of a possible 10,000). Were it not, we may have run more repeats, however, the experimental design would likely have to be changed to increase the unexpectedly low copying rate.

Second, what is the justification for presenting only two options when copying the answers (e.g., “Domain-Specific vs. Cross-Domain”)? I understand that this simplifies the task of the participant. However, the design becomes more complicated (to carry out, to report and to interpret) as we need to compare between pairs of conditions. Why is it not possible to present a list of options that convey all possibilities (Domain-Specific, Domain-General, Cross-Domain, Random Cue)? If there are good reasons to present only two options, I would like to read them in the manuscript.

Firstly, comparing two information choices allows us to understand the ‘hierarchy’ of prestige information, in that comparing two reveals that one specific option is chosen *only when there is no better alternative.* If we presented all four options at once, then, given our current results, it’s likely that participants would preferentially choose domain-specific information most of the time, but that would tell us nothing about if/when/why domain-general prestige is ever preferred (which it is, when domain-specific information is not available). Nor would it tell us anything about if/when cross-domain prestige is used (which it is, only when access to “higher quality” information such as domain-specific or domain-general information is unavailable). These two-choice comparisons thus help us to understand the relative use and preference of each source compared to the others. Secondly, presenting the two choices is simpler for participants, in that, presenting all at once would likely be a bewildering amount of information to interpret, and would take a lot of explanation ahead of time and comprehension checks to make sure participants understood the source of each of the four choices at once. Thirdly, it is much easier to appropriately analyse one choice out of two statistically, compared to conducting an analysis where the outcome variable is one choice out of four, so we can be more confident in our statistical analysis when only comparing the outcomes of two choices.

-The choice of this procedure/design also has consequences in terms of statistical power. Although the sample size is overall good, we have small cell sizes. This compromises the generalization of the results.

The cell sizes of interest aren’t small, as the primary measure we are interested in copying behaviour in Round 2, we had between 200-700 instances of copying in Round 2 of *each condition* (see Fig.4), resulting in 1,945 observations in total. This is because each participant had 100 copying opportunities, and we recruited 100 participants per condition. Our analyses reflect this hierarchical structure and account for the fact that each participant contributes multiple and varied numbers of copying instances per group and per condition.

-Additionally, and related to the previous point, I would like to see a justification for the sample size: if it was not decided a priori, a sensitivity analysis would suffice, to have an idea about the smallest effect that your study could detect with reasonable power. Choosing a sample size similar to a previous study is generally not a good strategy to decide the N.

We don’t use effect sizes as our analyses are Bayesian, so we are not interpreting whether an “effect” is significant (which requires power analysis), nor are we interested in an “effect” of an experimental manipulation, but are interested in participant’s tendencies and preferences for information that arises from their own behaviour within a learning scenario. We have interpreted our results on the probability scale (Fig 3), not on the scale of the model estimates, showing that participants were choosing the predicted information in over 80% of their choices. Our experiment contained 47,313 observations, of which 5,385 were copying choices, instances that occurred naturally in randomly assigned groups of online participants. Out of these natural copying choices we had 1,945 observations of interest (copying in the second round), which we feel is an ample number of observations.

-The authors use a variety of domains to test the generalizability of their results. As the authors discuss (in the Discussion), the correlation between domains that participants assume can be important. Could this be a problem when interpreting the current data? For example, if participants believe that the skills necessary to succeed in two of the tasks are similar, or the same (e.g., both Geography and Language are based on general knowledge, therefore the ones succeeding in Geography should do fine in Language), then they might show apparent preference for general domain prestige. I wonder if the results could be different depending on the set of domains used in the experiment: whether they are closely related or they are completely independent (e.g., a pshysical task and an intellectual task). Perhaps this could be added to the Discussion.

Whether domains are “closely related or completely independent” is an empirical question based on how correlated success on each domain is, and whether success on each domain is determined by some underlying trait such as general intelligence etc. Furthermore, people’s intuitions as to how correlated two domains are (e.g. ‘intellectual’ versus ‘physical’) relies on the extent of their experience and understanding of those domains as separate or related. We feel we discuss these points extensively in three separate paragraphs, lines 714 - 787 of the discussion. These paragraphs relate to: 1) people’s perceptions and intuitions about how correlated different domains are 2) people’s experiences of different domains meaning a domain-general model for one individual might appear as a domain-specific model for someone else with less experience in the general domain (with the example of science) and 3) that correlations between domains may depend on whether some underlying trait contributes to success in both domains. All three points address why comparing an assumed ‘intellectual’ task to an assumed ‘physical’ task could lead to more or less domain-specific copying, depending on who is doing the copying and who is being copied, and is an open question.

More specific comments:
-I would like to see additional information about what participants see on the screen. For example, when they choose to “ask someone else”, what information is then presented? We have screenshots for the questions, but not for these informative screens.

We have now included an example screenshot of a participant viewing the prestige choices and the subsequent participant choices as part of Figure 1.

-Is it possible to name the conditions in a more informative way than “condition A, condition B...”? I had to check the Table multiple times to remember which cues are being contrasted in each condition.

We now refer to the conditions by the type of prestige cue they are comparing, with their original condition in brackets. Hopefully this makes the results section easier to follow.

-I think that describing in the paper the function used to compute the Pearson coefficients (“cor.test()”) is unnecessary. Is there anything in the way this function works that is important or unusual so that it deserves comment?

We have now removed reference to this specific function as it is available in the analysis scripts for interested readers.

-I am curious as to why the credible intervals have a width of 89%. I get that the popular option of 95% (at least it is more popular in frequentist analyses) means less stable estimations. By then, why not 90%? I don’t mean that the manuscript or the analyses are wrong, but the choice of 89% had me wonder, and there is no justification in the text.

We have added the justification that the choice of 89% credible intervals is to prevent readers from assuming that the interval should be interpreted in the same way as 95% confidence intervals and performing unconscious significance tests inappropriately.

**Reviewer #2:**

Introduction

- It is necessary to provide a complete definition of prestige. In this vein, it would be interesting to clarify if there is any connection between source prestige and source credibility, commonly identified to consist of expertise and trustworthiness (for a review, see Pompitakpan, 2004) or other attributes, such as age or gender.

Thank you for taking the time to carefully read and comment on our manuscript. We have now added a more detailed introduction to prestige and include this reference when referring to other papers that review prestige as well.

- Please, provide an explanation of the difference between prestige information and direct success information (line 67).

We have now altered the wording of the introduction to hopefully make clearer what we mean by prestige information and direct success information.

- Please, justify the reason why it is important to better understand domain-specificity and -generality of prestige-bias. Which kind of implications might these two kinds of prestige bias have in real life? It could be very helpful to add some specific examples.

We have now added more explicit examples of when domain-specific and domain-general prestige interact in real life, such as famous sportspeople giving political or medical advice outside of their domain-specific expertise. (Line 80).

Method_Procedure

In general, the information provided in this section is incomplete or not clear. In several cases, it is necessary to consult previous studies of the authors to fully understand the content. For instance:

- What is the reason why if everyone chose to “Ask Someone Else” then participants were shown a message saying “sorry, everyone chose to ‘ask someone else’ so no one can score points for this question?

The reason is because if everyone chose to copy for a single question, then there wouldn’t be any participants’ answers to copy from for that given question, as no one answered the question themselves. We’ve now clarified this wording (lines 189-202).

- The step “They then chose a demonstrator whose answer they used for that question (lines 207-208)” is confusing. Could you offer a more detail explanation?

Thank you for pointing this out, we have now clarified this section so hopefully it is clearer (now lines 189-202).

Discussion

- Please, explain in more depth the implications the ‘hierarchy’ of prestige detected might have in real life.

We have now added a section to the discussion to give a real world example of how this prestige hierarchy might play out (lines 753-758), as well as the general implications of our main result (line 727-730).

Minor comments

7. Line 470: There is a double comma.

Thank you for spotting this, it has now been deleted.

Reviewer #3:

This is a well-written manuscript on an interesting topic for evolutionary psychology with sound methods and clean results. The pre-registration is also sound and the protocol is followed carefully by the authors. It is true that probably the results are not smashing but this is not a concern in PLOS One (and not a concern in general for me). Therefore, I can just congratulate the authors. If I am not wrong, this is the first time I recommend acceptance of a manuscript in the first revision round.

I have just one very minor comment: it would be interesting to test whether participants choose to copy those who are most copied when they can choose to copy based on scores; that is, what of these two information cues is preferred when are both present? Unfortunately, as far as I understand, the current design does not allow for this analysis and should be left for future research. Maybe the authors want to discuss this point.

Antonio M. Espín

Thank you so much for your kind review. To address your minor comment, we did in fact test whether participants chose prestige when success information was available in the previous experiment that was the foundation for this experiment, found here: <https://www.nature.com/articles/s41598-020-68982-4>
