# Supplementary material for: Trusting the experts: The domain-specificity of prestige-biased social learning
Source: PLoS One. 2021 Aug 11;16(8):e0255346. doi: 10.1371/journal.pone.0255346 (PMC8357104; doi:10.1371/journal.pone.0255346)
Supplement: Attachment — Submitted filename: response_reviewers_120721.docx [file pone.0255346.s004.docx]

==============================

I think that the ms is essentially ready for publication. Before accepting it, I'd like the authors to consider the following changes:

lines 60-62 "Crucially, prestige-bias only evolved, and is only adaptive, because the prestige was first acquired due to success..." -> This sounds like a proven fact ("only evolved") but it is actually speculation based on cultural evolutionary theory of prestige. Consider changing the beginning of the sentence to "According to the cultural evolutionary theory of prestige..." and then remove "Consistent with this cultural evolutionary theory of prestige" in the following sentence.

lines 146-148 In "As before, we use participants..." consider rewriting as "As before, instead of manipulating prestige experimentally, we use participants..."

==============================

Thank you again for engaging constructively with our work. We have happily made these suggested changes and are happy to resubmit.
